# Relaxing the import proportionality assumption in multi-regional input–output modelling

**DOI:** 10.1186/s40008-021-00250-8

**Published:** 2021-10-09

**Authors:** Simon Schulte, Arthur Jakobs, Stefan Pauliuk

**Affiliations:** Industrial Ecology, University of Freiburg, Tennenbacher Str. 4, 79110 Freiburg, Germany

**Keywords:** Import proportionality assumption, Uncertainty, Environmentally-extended multi-regional input–output, Carbon footprint, Land footprint, Water footprint, Material footprint, Footprint analysis

## Abstract

In the absence of data on the destination industry of international trade flows most multi-regional input–output (MRIO) tables are based on the import proportionality assumption. Under this assumption imported commodities are proportionally distributed over the target sectors (individual industries and final demand categories) of an importing region. Here, we quantify the uncertainty arising from the import proportionality assumption on the four major environmental footprints of the different regions and industries represented in the MRIO database EXIOBASE. We randomise the global import flows by applying an algorithm that randomly assigns imported commodities block-wise to the target sectors of an importing region, while maintaining the trade balance. We find the variability of the national footprints in general below a coefficient of variation (CV) of 4%, except for the material, water and land footprints of highly trade-dependent and small economies. At the industry level the variability is higher with 25% of the footprints having a CV above 10% (carbon footprint), and above 30% (land, material and water footprint), respectively, with maximum CVs up to 394%. We provide a list of the variability of the national and industry environmental footprints in the Additional files so that MRIO scholars can check if an industry/region that is important in their study ranks high, so that either the database can be improved through adding more details on bilateral trade, or the uncertainty can be calculated and reported.

**Supplementary Information:**

The online version contains supplementary material available at 10.1186/s40008-021-00250-8.

## Introduction

Multi-Regional input–output modelling (MRIO) is widely applied to study the relationship between economic activities and their upstream environmental, social and economic impacts (Miller and Blair 2009; Wiedmann 2009). To do so, MRIO links national inter-industry accounts with international trade data (Tukker et al. 2018). Although extensive inter-regional trade information is available, they often lack the required level of detail needed to compile MRIO tables without having to rely on strong assumptions (Rodrigues et al. 2016; Dietzenbacher et al. 2013).

MRIOs are commonly build from rectangular Multi-Regional Supply and Use Tables (MRSUTs) which are then converted to symmetric MRIO tables (see Eurostat 2008 for more details). The compilation of MRSUTs requires information on the use (or consumption) of product *i* from region *a* in the target sector *j* in region *b* for all regions, products and target sectors covered by the MRSUT. The target sector can either be an individual industry (intermediate consumer) or a final consumer (typically distinguishing households, government and capital formation). However, data on imported inputs are not available at the level of individual industries and final demand categories but by “Broad End-use Category” BEC (OECD 2020). As the name implies the BEC classification system only broadly distinguishes between intermediate consumption, household consumption, capital goods and mixed end-use (products where the end-use is unclear e.g. cars can be purchased both for household consumption and as capital goods).

In effort to overcome this lack of information, MRIO/MRSUT compilers proportionally distribute the imported commodities over the target sectors in the importing region so that an exporting region’s share of the total import volume of a product is the same for each target sector (see Fig. 2D). This assumption is often referred to as the “proportionality assumption” (Rodrigues et al. 2016; Peters et al. 2011). The proportionality assumption underlies all current global MRIO tables, however the level at which the proportionality assumption is applied varies. EXIOBASE (Stadler et al. 2018), Eora (Lenzen et al. 2013) and GTAP (Peters et al. 2011) for instance allocate imports to the target sectors without the differentiation in intermediate use, consumption, and capital (i.e. not using BEC data) thus using the same proportions for *all* target sectors be it industries or final consumers. WIOD, by contrast, take the BEC data to distribute imports to aggregate end-use sectors but then also use the same proportions for all industries and final demand categories, respectively (Dietzenbacher et al. 2013). One reason why only WIOD uses the BEC data is that the BEC data by no means covers all industries, countries and years at the resolution required to produce such high resolution MRIOs as EXIOBASE or Eora.

Although the assumption that imports are distributed proportionally among individual industries and end-consumers might provide a practical solution to the lack of more detailed data, the assumption might be flawed for several reasons, possibly resulting in biased MRIO-based results. In the following, we first address some aspects that could lead to ‘real’ import shares differing significantly from the proportionality assumption. We then work out the conditions which additionally must be fulfilled so that assuming proportional import shares in such situations leads to a bias in the MRIO results.

### Why the proportionality assumption might bias MRIO-based footprints

One reason why the proportionality assumption might be flawed is the aggregation bias: due to the aggregation of firms to broader industry sectors, one such a sector might include rather heterogeneous products (in terms of physical properties and/or prices) (Majeau-Bettez et al. 2016).

In EXIOBASE for example, raw unfabricated leather products and luxury leather handbags are aggregated into one “Leather and leather products” sector (Stadler et al. 2018). Imagine three countries: Country *a* and *b* both export leather and leather products to country *c*. However, *a* is specialised in raw leather, while *b* exports mainly luxury leather handbags. In country *c* two industries *i* and *j* use imported leather, however, sector *i* buys raw leather from country *a* (to further process it), while sector *j* imports luxury leather handbags from country *b* (to retail them). However, since this knowledge on the exact use of imports is not available for MRIO compilers, they assume that for both industries *i* and *j* the shares of imported leather products coming from country *a* and *b*, respectively, are the same.

Depending on the application case this assumption of proportional import shares could lead to biases in the outcome of MRIO studies if further conditions are met: If a MRIO is used to study the environmental implications at the level of industry or product sectors [e.g. study the footprint of sector *i* (Wiedmann et al. 2009; Huang et al. 2009), or of a consumption basket (Hardadi et al. 2020; Ivanova et al. 2016)], the proportionality assumption biases the outcome when there is a large variation in the impact intensities between the products of the exporting countries. Presumably, the cheap raw leather products from country *a* have a higher carbon emission intensity (i.e. emissions per Euro) than the expensive luxury leather handbags from country *b*. Hence sector *i*’s carbon footprint would be underestimated because the upstream emissions related to *i*’s use of imported leather products are averaged among both importing countries, instead of taken from the carbon intense leather sector of country *a*.

If a MRIO practitioner is interested in the environmental implications at the national level (e.g. Schmidt et al. 2019), a large variation in the impact intensities between the products of the exporting countries, however, is not yet sufficient to lead to a bias in the results. Then additionally the products of industries *i* and *j* have to differ in terms of what proportion is consumed domestically and what proportion is exported. If industry *i* (importing from *a*) mainly produces for export, while *j*’s luxury leather handbags are mainly for the domestic market, the proportionality assumption would lead to an overestimation of *c*’s national carbon footprints because the upstream emissions related to industry *j*’s use of imported leather products would partly incorporate emissions which are actually linked to *i*’s leather imports (which should not show up in the footprint of country *c* since *i* produces for export).

There exist also other reasons why imports might not be distributed proportionally among target sectors. These are, for example, geographical reasons, e.g. the location of firms from one sector closer to large harbours or towards the border to a neighbouring country, or historically grown trade relations between firms in different countries.

### Literature review, research gap and research question

Even though several MRIO compilers mention the potential problems related to the proportionality assumption (Stadler et al. 2018; Peters et al. 2011; Lenzen et al. 2013; Dietzenbacher et al. 2013), so far only few studies approached the problem quantitatively. Puzzello (2012) investigated the effect of the proportionality assumption on the factor content (capital, labour, services, ...) of trade. The author compared the results of two different methods to compile the Asian MRIO table, one assuming proportional import shares, and another survey-based including bilateral details on trade. She foundinds the net bias introduced by the proportionality assumption to be small “only because the biases on exports and imports of factor services cancel each other out”.

In their working paper Milberg and Winkler (2010) studied the error from the proportionality assumption on the estimate of the effect of offshoring on the German labour demand for 36 sectors. They estimated the effect of offshoring applying the same econometric model but with two distinct data sets: one based on the proportionality assumption and one with additional details on the use of German imports. They found a large difference in the regression coefficient estimates between the two versions, in many cases even with reversed signs.

Feenstra and Jensen (2012) did a similar comparison for the estimates of material offshoring from US manufacturing. They calculated the shares of imported intermediate inputs of individual manufacturing sectors in two ways: (1) using firm-level data on imports and production and (2) applying the proportionality assumption. They found a moderate to high correlation between the offshoring shares calculated with the distinct methods (correlation coefficient of 0.68 and 0.87 if shares are value weighted).

Recently, Jiang et al. (2020) compared the material footprints of China and Chinese provinces based on two approaches: one with the assumption of proportional provincial import shares, and one with the inclusion of detailed data on Chinese inter-provincial trade. They found that the Chinese national material footprint is not significantly influenced by the choice of methods. Across provinces, however, the authors found variations in the material footprints between the two methods in the range from − 9% to + 14%. For disaggregated materials the differences between both methods were even ranging between − 48% and + 34%.

From the literature review we identify two major research gaps. First, all studies so far are region-specific, i.e. they only examine how the calculated impacts change when including bilateral trade details for *one* country/region. Second, all but one study investigate economic effects, with only Jiang et al. (2020) considering an environmental impact indicator. The effect of the proportionality assumption on other environmental indicators, such as carbon, land or water footprints, however, have not been investigated so far. Which is surprising given that environmental footprinting has been a major field of application of MRIOs in the last decades (Wiedmann 2009; Wiedmann et al. 2015; Li et al. 2017; Brizga et al. 2017).

Against this background, we test how sensitive environmental footprint estimates are towards changes in the allocation of import flows not only for individual regions but for the entire world trade. Since we do not know the true import shares we randomise the allocation of import flows to different target sectors while keeping fixed the available information, namely (i) the total imports per country and product, and (ii) the total use of product import per target sector. The aim of our study is to quantify the maximum uncertainty introduced by the proportionality assumption. We focus on the four most commonly used types of environmental footprints (carbon, material, land, and water) on two levels (country and industry).

## Material and methods

To quantify the uncertainty of environmental footprints introduced by the proportionality assumption we undertake the following steps: We generate 4897^1^ MRIO tables with globally randomised import allocations. With each of these new MRIO tables we then calculate national and industry footprints and investigate the variability of these footprints.

### Data

We use EXIOBASE (Version 3.4), a global MRIO database that is based on the proportionality assumption and is widely used for environmental footprint analysis (Stadler et al. 2018). We use the tables for the year 2011 in current prices in its original resolution covering 163 industries in 44 countries and 5 Rest of the World (RoW) regions. EXIOBASE provides MRIOs in two different versions which differ in the model used to create a symmetric MRIO table from a rectangular MRSUT (Eurostat 2008): industry-by-industry tables based on the fixed product sales assumption covering 163 industries, and product-by-product tables based on the industry technology assumption covering 200 products. Product-by-product MRIOs offer a higher level of detail for (trade) transactions than industry-by-industry MRIOs as the former distinguish between different types of coal, natural gas, coke, refined products, biofuels and gas distribution services. However, this additional level of detail comes at the expense of a longer computation time for calculating environmental footprints. Since the computation time of a matrix inversion that is required when calculating environmental footprints (see below) scales exponentially with the number of dimensions (Çetinay et al. 2020), and computation time was constrained to 40 h by the cluster used for the analysis, we decided to work with the industry-by-industry tables. This allows us to perform considerably more simulations and thus to get more robust results. We believe that the choice of model does not significantly change our conclusions, since as stated by Eurostat in their Manual of Supply, Use and input–output Tables under most circumstances “industry-by-industry tables [are considered] a good approximation of product-by-product input–output tables” (Eurostat 2008).

### Generating a MRIO table with globally randomised import allocations

We first show our approach to randomise the allocation of the imports of the output of *one* industry to the target sectors in *one* country. To generate an entire new MRIO table with *globally* randomised import allocations this procedure has to be repeated for *all* industries and countries covered by the MRIO.

**Fig. 1:**
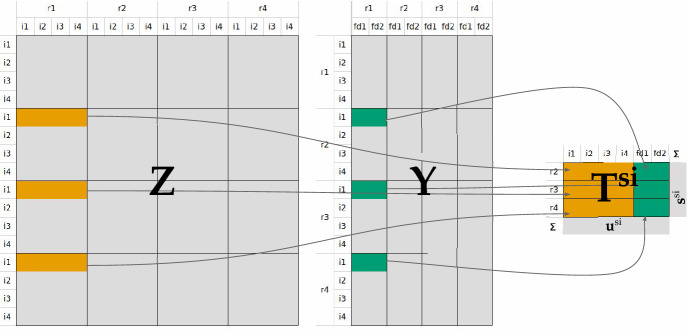
A simplified MRIO system with four regions, four industries and two final demand categories to exemplify how the import matrix ${\mathbf {T}}^{si}$ is extracted from the inter-industry matrix ${\mathbf {Z}}$ and the final demand matrix ${\mathbf {Y}}$

We refer to the sets of exporting regions as *R*, exporting industries as *I*, importing regions as *S* and importing target sectors (comprising industries and final demand categories) as *J*. Matrices (capital letters) and vectors are represented as bold characters. For demonstration in this paper, we use a simple industry-by-industry MRIO system with four regions, four industries and two final demand categories to exemplify how the imports of the output of industry $i \in I$ (say “leather industry”) from the exporting countries *R* are randomly allocated to the six target sectors *J* of region $s \in S$ (say Germany) (see Fig. 1). Since the proportionality assumption concerns only the inter-industry matrix ${\mathbf {Z}}$ and the final demand matrix ${\mathbf {Y}}$ at that stage we omit the other elements of typical environmentally-extended MRIO tables (primary inputs, total output, environmental extensions).

The problem we are facing is the allocation of the import flows of a given good (here: the output of industry *i* = leather industry) from different countries *R* to different target sectors *J* in a given country (here: *s* = Germany), where both (i) the total amount of imports by each exporting country $r \in R$, and (ii) the total amount of imported inputs for each target sector $j \in J$ are known. This problem can be represented in the form of a matrix (the “import matrix” ${\mathbf {T}}^{si}$), where both the row sums ${\mathbf {s}}^{si}$ (= imports of the output of industry *i* to region *s* by region of origin *R*) and column sums ${\mathbf {u}}^{si}$ (= inputs of industry *i*’s output by target sector *J* in region *s*) are known, but cell entries are not. Formally expressed we know thus:

1 $$\sum _{j\in J}t^{si}_{rj}= \mathbf {s}^{si}$$

2 $$\sum _{r\in R}t^{si}_{rj}= \mathbf {u}^{si}$$

Figure 1 shows how we extract the import matrix ${\mathbf {T}}^{si}$ from the inter-industry matrix ${\mathbf {Z}}$ and the final demand matrix ${\mathbf {Y}}$. For the sake of readability, we omit the superscripts in the following. Summing ${\mathbf {T}}$ *row-wise* we get the vector of import flows ${\mathbf {s}}$ depicting the imports (supply) of the leather industries’ output from different exporting regions to Germany (Eq. 1). Summing ${\mathbf {T}}$ *column-wise* we get the vector ${\mathbf {u}}$ depicting the use of the imported leather industries’ output in different industries and final consumption categories in Germany (Eq. 2).

Now, the aim is to randomly allocate the region-specific supply ${\mathbf {s}}$ to the industry-specific use ${\mathbf {u}}$. Thus, we want a ‘new’ randomised import matrix ${\mathbf {T}}'$. We follow the compilers of the most prominent global MRIOs EXIOBASE (Stadler et al. 2018), Eora (Lenzen et al. 2013) and GTAP (Peters et al. 2011) and do not—unlike WIOD (Dietzenbacher et al. 2013)—include information on the BEC.

**Fig. 2:**
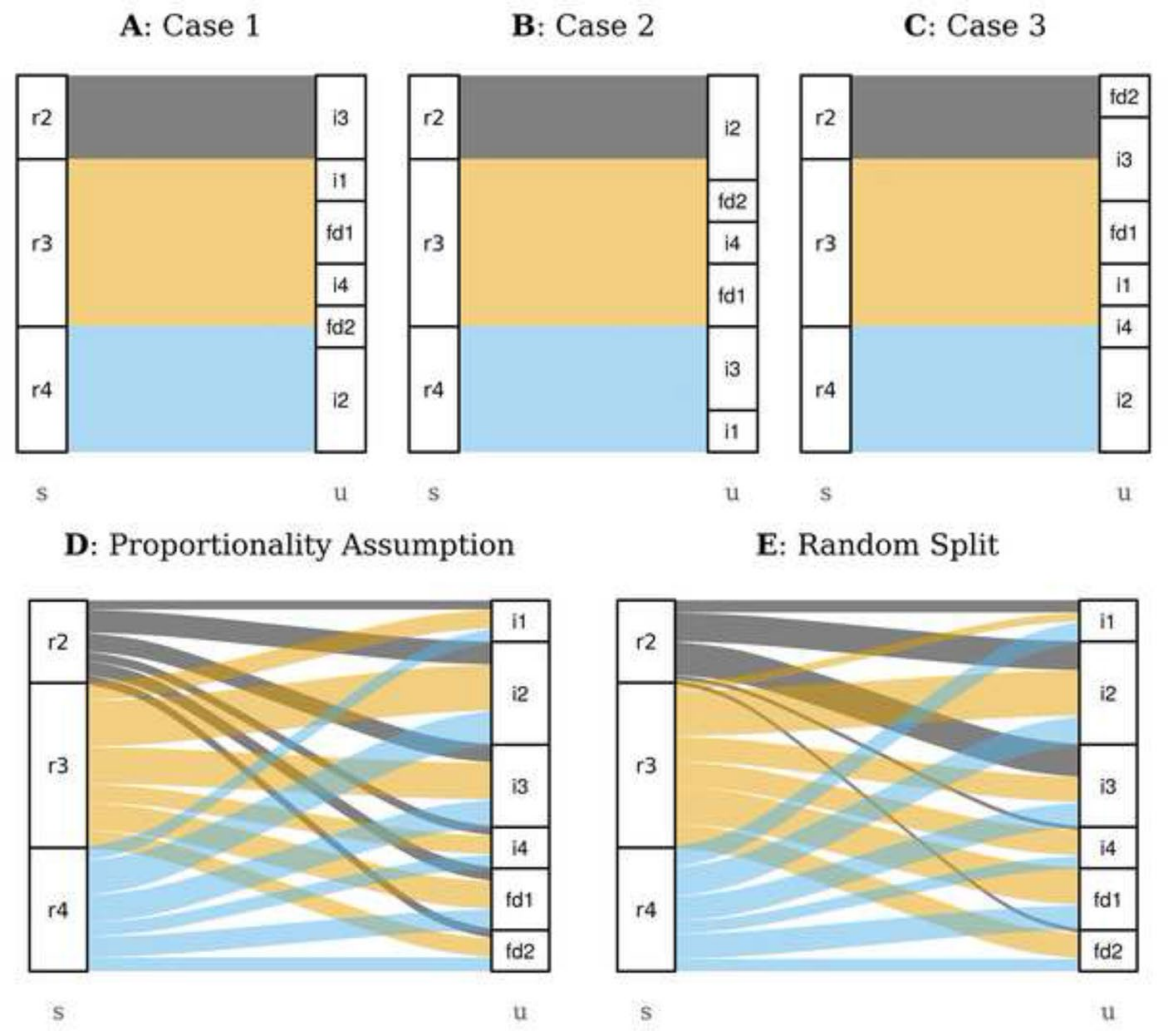
Sankey diagrams illustrating different possible ways to allocate ${\mathbf {s}}$ to ${\mathbf {u}}$. **A**–**C** Three possible outcomes of our algorithm. **D** allocation based on proportional import shares. **E** An example of an intermediate case which is not covered by our algorithm

We apply an algorithm to randomly allocate ${\mathbf {s}}$ to $\mathbf{u}$ block-wise which works as follows (see Fig. 2A–C, a pseude-code version of the algorithm can be found in Additional file 1).

- Step 1: We start by taking the supply of region 1 ($s_1$ = 1st element of ${\mathbf {s}}$) and the use of a randomly chosen target sector *j* ($u_j$ = *j*th element of ${\mathbf {u}}$).
- Step 2: Now we differentiate three cases: If the supply from country 1 equals or is smaller than the use of industry *j* (*case 1* or *2*, respectively) we allocate the entire supply of country 1 to industry *j*. If, however, the supply of country 1 is larger than the use of industry *j* (*case 3*), the fraction of country 1’s supply which equals the entire use of *j* is allocated to *j*. In Fig. 2A–C these three cases are illustrated.
- Step 3 is depending on which case has occurred in the previous step:
  - In case 1, both the entire supply of country 1 and the entire need of industry *j* have been accounted for. Thus, we can go over to the next country 2 and compare its supply with the next randomly chosen industry following the procedure described under step 2 and 3.
  - In case 2, the entire supply of country 1 has been accounted for but not the need of industry *j*. Thus, we go over to the next country 2 and compare its supply with the remainder of industry *j* following the procedure described under step 2 and 3.
  - In case 3, the entire need of industry *j* is met, but country 1 still has imports left. Therefore, we continue with the next randomly chosen industry (in our example $i_3$) and compare its need with the remainder of country 1’s supply following the procedure described under step 2 and 3.
  We run this algorithm until the supplies of all countries have been accounted for and the needs of all industries have been met. This condition will certainly be reached since all trade flows in MRIO tables are balanced, i.e. the total imports of industry *i*’s output into region *s* equals the total use of imported inputs ($\sum _{r}s_{r} = \sum _{j}u_{j}$).

Carrying out the above outlined procedure for the imports of each industry output $i \in I$ into each region $s \in S$ results in ‘new’ matrices ${\mathbf {Z}}^{\text{new}}$ and ${\mathbf {Y}}^{\text{new}}$ where all imported industry outputs into all countries are randomly allocated to the target sectors while keeping fixed both, (i) the total imports per country and industry output, and (ii) the total use of product import per industry sector.

Our approach is strictly speaking not a randomisation, since we do not consider all possible versions of the import matrices. In the lack of knowledge on bilateral trade details we should have to assume that each of the theoretically infinite possible versions of this import matrix is evenly likely. However, with our algorithm we only consider the extreme versions of the import matrix. With “extreme” we mean that our algorithm produces import matrices where imports are bundled and allocated block-wise to the target sectors (Fig. 2A–C). Thus, we miss all versions of the import matrix ${\mathbf {T}}$ where the import flows from different regions are split and randomly distributed over a large number of target sectors (i.e. all target sectors import a bit from country *a*, a bit from *b* and so on, Fig. 2E).

So instead of randomly sampling out of *all possible* versions of the import matrix, we only sample out of *all extreme* ones. Given the number of repetitions to be limited by computational issues—in our case to 4897 repetitions—with our approach we increase the probability to capture the extreme ends of the “real” distribution of the uncertainty of the respective footprint. Thus, we come closer to an estimate of the *maximum possible* uncertainty of the respective footprint which is the aim of our study.

### Calculating environmental footprints

We calculate the four most used environmental footprints: carbon, land, material and water (Steinmann et al. 2018). Following Steinmann et al. (2018) we define these footprints as the consumption-based ...

- ... emissions of the greenhouse gases $\text {CO}_2$, $\text {CH}_4$, $\text {N}_2$O, $\text {SF}_6$, hydrofluorocarbons (HFC), and perflourocarbons (PFC) weighted by their global warming potential based on a time horizon of 100 years (Myhre et al. 2014) (carbon footprint)
- ... area of land required by forestry, agriculture, infrastructure, etc. (land footprint)
- ... mass of all used extractions including metal ores, other minerals, wood, fish, and crops (material footprint)
- ... volume of the total blue water consumption (water footprint).

To calculate the environmental footprints at the national and industry level we use ${\mathbf {Z}}^{\text{new}}$ and ${\mathbf {Y}}^{\text{new}}$, along with the stressor matrix ${\mathbf {S}}$ containing the relative environmental impacts per unit of sector output, the output per sector ${\mathbf {x}}$, the characterisation matrix ${\mathbf {C}}$ that weights the environmental impacts according to the four footprints, and the matrix of direct impacts from final demand ${\mathbf {H}}$ storing the total direct impacts caused by all final demand categories in a region by footprint category, all provided by EXIOBASE (Stadler et al. 2018; Miller and Blair 2009).

We first calculate the Leontief inverse matrix ${\mathbf {L}}$ as

3 $${\mathbf {L}} = ({\mathbf {I}}- {\mathbf {Z}}^{\text{new}} {\hat{\mathbf{X}}^{-1}}),$$

where ${\mathbf {I}}$ is the identity matrix and ${\hat{\mathbf{X}}^{-1}}$ is a square matrix with $1/x_i$ on the main diagonal and zeros elsewhere.

We then calculate the matrix of environmental multipliers $\mathbf{E}$ storing the environmental impacts per Euro of final demand produced by industry sector:

4 $${\mathbf {E}} = \mathbf {CSL}.$$

What we refer to as industry footprints ${\mathbf {F}}^{\text{ind}}$ we obtain by multiplying the environmental multipliers with the amount that is finally demanded for each industry’s output:

5 $${\mathbf {F}}^{\text{ind}} = {\mathbf {E}} {\hat{\mathbf {Y}}},$$

where ${\hat{\mathbf{Y}}}$ is a square matrix with ${\mathbf {y}} = \sum _{j}y_{ij}$ (i.e. the sum of final demand over final demand categories *j*) on the main diagonal and zeros elsewhere.

National footprints ${\mathbf {F}}^{\text{nat}}$ we calculate as

6 $${\mathbf {F}}^{\text{nat}} = {\mathbf {E}} {\mathbf {Y}}^{\text{new}} + {\mathbf {H}}.$$

We carry out 4897 simulations runs, thus resulting in samples of 4897 different carbon, water, land and material footprints at national and industry level respectively. We quantify both the absolute and the relative variability within these samples. To measure the *absolute* variability we use the Standard Deviation (SD), while for the *relative* variability we use the Coefficient of Variations (CV) defined as

7 $$\text{CV} = \frac{\text{SD}}{\mu},$$

where $\mu$ is the sample’s mean.

We choose the more commonly used SD and CV instead of a measure that is more robust against outliers such as the (relative) Median Absolute Deviation, so that we can compare our results with other studies on the uncertainty of environmental footprints. In Additional files 3 and 4 we also provide our results with these alternative measures of variability. Since both SD and CV do not give any information about the exact appearance of a distribution (e.g. its skewness, number of modes, etc.), we take a closer look at the sample distributions for some example industries/nations by looking at their probability density function and describing their variability in terms of their 2.5th and 97.5th percentiles.

## Results

We present the sensitivity of different environmental footprints when relaxing the proportionality assumption on two different levels: first, at the level of nations, and second, at the level of industries.

### National footprints

**Fig. 3:**
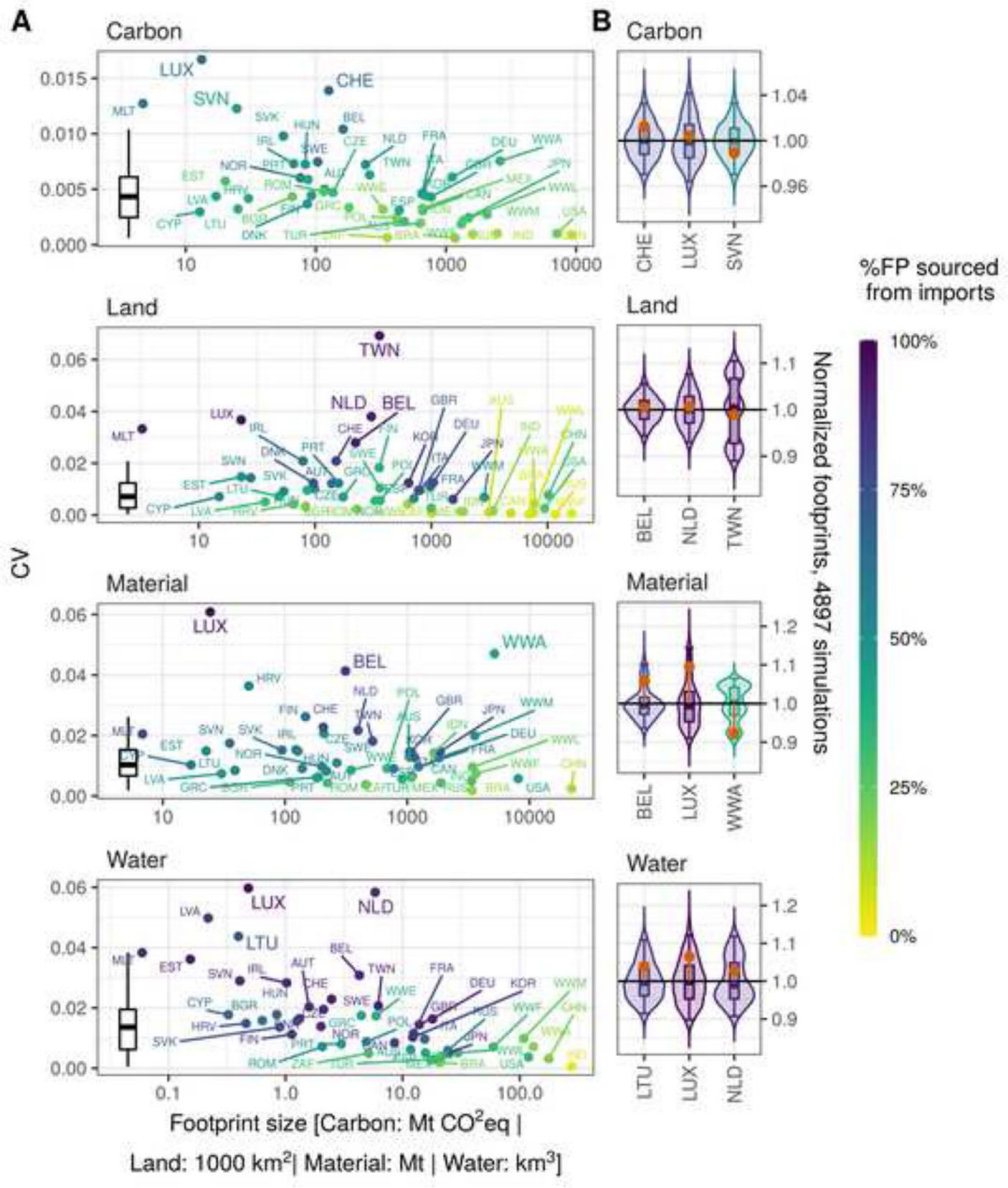
**A** The relative variability (CV) of the national footprints compared to their absolute size assuming proportional import shares. Boxplots show the distributions of the CVs across the 49 regions to facilitate comparison between the different types of footprints. Country codes according to ISO 3166-1 alpha-3 except RoW regions (see Additional file 5). **B** The sample distributions of the national footprints exemplary for some selected interesting regions. The footprints where normalised by dividing each sample by the mean of all 4897 samples. The violin plots show the probability density and the range of all samples. The boxplots show the inter-quartile range (IQR) where 50% of all samples are situated (boxes), the sample’s median (horizontal line) and the range from the 2.5th to the 97.5th percentile (whiskers). The red points show the footprints size assuming proportional import shares, also normalised by the mean of all samples

Figure 3A shows the relative variability (CV) of the national footprints compared to the absolute size of the footprints assuming proportional import shares. The points and country labels are coloured by the percentage of the footprint which is sourced from imports.

Overall, the CV of the carbon footprints is lower on average compared to the other three footprint categories (boxplots). A high CV is mostly associated with regions with a small absolute footprint size and and a high import share. The highest CV in carbon, material and water footprints can be seen in Luxembourg (LUX) with values of approximately 0.02 (carbon) and 0.06 (material and water). Taiwan (TWN) shows the highest relative variability for the land footprint with a CV of close to 0.07. The percentage of these footprints sourced from imports is 66% (LUX, carbon), 95% (TWN, land), and 99% (LUX, material and water). Other regions of interest with a relatively high CV and a relevant footprint size are—in the case of carbon footprints—Switzerland (CHE) and Slovenia (SCN) with CVs of 0.01 each. In the case of land footprints, regions worth mentioning are the Netherlands (NLD, CV of 0.04) and Belgium (BEL, CV of 0.03), which both have a high population density and are highly dependent on imports of land-intensive food products and materials. RoW Asia and Pacific (WWA) stands out when looking at its material footprint having the third largest absolute material footprint with more than 5000 Mt and the second highest CV with a value of 0.05. In the case of water footprints, interesting regions are again the Netherlands (CV of 0.06) and, less pronounced, Lithuania (LTU, CV of 0.04).

Figure 3B shows the distribution of the 4897 simulated national footprints exemplary for some selected regions (the regions mentioned earlier). For distributions of all national footprints please refer to in Additional file 2: Figure S1. The footprints where normalised by dividing each sample by the mean of all 4897 samples.

The 95% confidence interval (CI) of Luxembourg’s carbon footprint ranges between ± 4% around the mean. Both, Luxembourg’s material and water footprint distributions are skewed towards higher values with a range between − 9% and + 14%, and − 10% and + 12%, respectively. Taiwan’s land footprint ranges between − 11% and + 10% around the mean (95% CI). Taiwan’s land footprint and WWA’s material footprint both show multi-modal distributions, i.e. they have several local maxima in their density function.

The difference between the footprints using the proportionality assumption and the sample mean, is particularly pronounced for the material footprints of Luxembourg (+ 10%), WWA (− 8%) and Belgium (+ 6%). The three countries exemplary listed with their water footprint show a difference around + 3 to + 6%, while the countries listed with their carbon and land footprints show only a comparably smaller deviance.

**Fig. 4:**
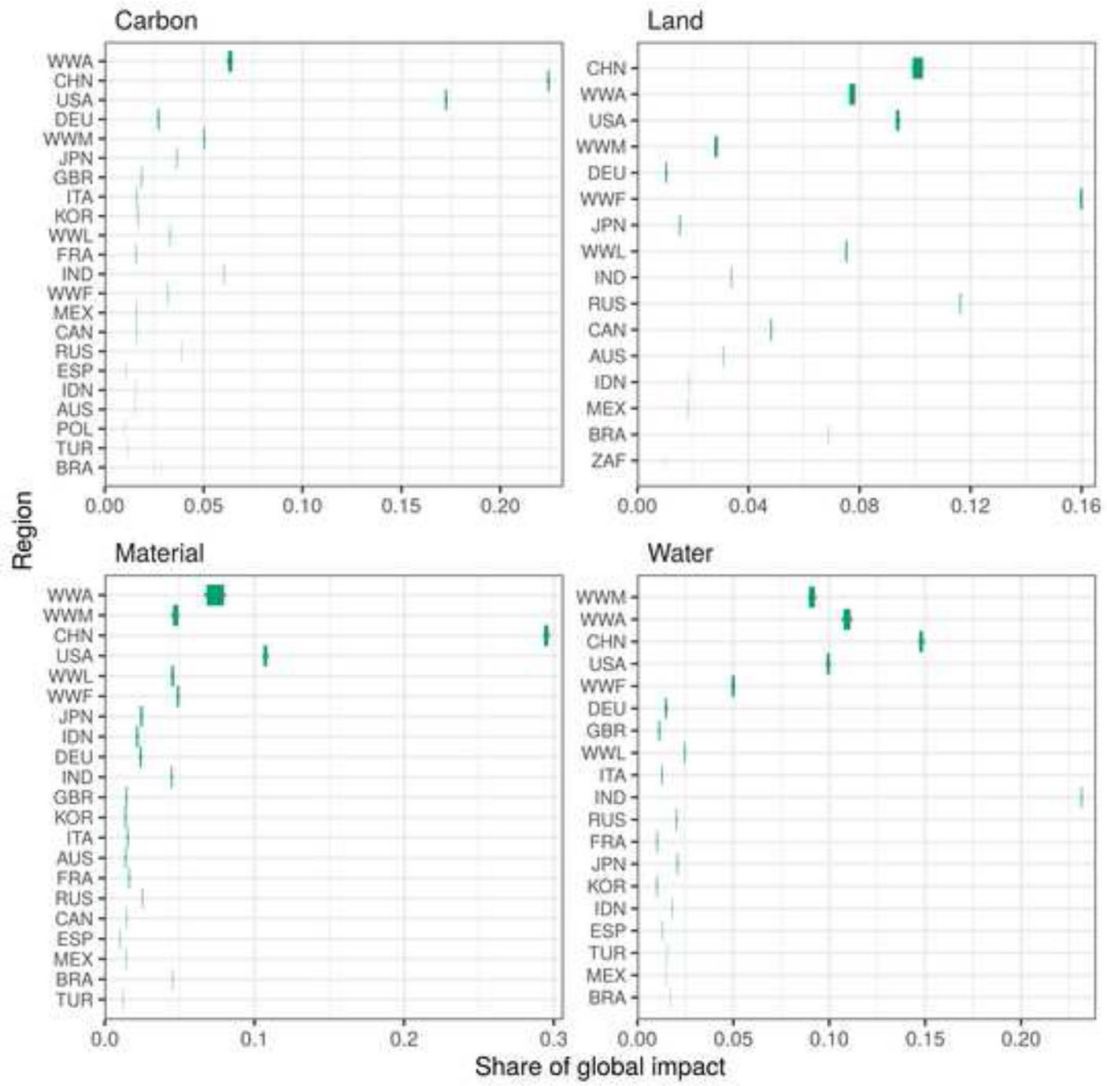
The absolute variability of the national footprints expressed as variability in the contribution to the global total impacts. The bars cover the range between the 2.5th and 97.5th percentiles. The grey whiskers extend to the min/max of the sample. The regions are ordered by their SD from top (high) to bottom (low). All regions with a share of less than 1% of the global impact are not included in the figure

Using Fig. 4, the relevance of the identified variability can be assessed against the background of the relative contribution of a region’s footprint to global impacts. The regions in Fig. 4 are ordered by their standard deviation (SD) from top (high) to bottom (low). WWA stands out with the highest SD regarding carbon, material and water footprints and the second highest SD regarding land footprints. China (CHN) has the highest SD in terms of land footprint and the second (carbon) and third (material, water) highest variability for the remaining footprints. Except WWA’s material footprint all previously discussed regional footprints with high relative variability have negligible variability in absolute terms. Overall, we find that the proportion of a region’s footprint in global impacts is relatively robust with regard to a relaxation of the proportionality assumption. Even for WWA’s material footprint which has the highest absolute variability the contribution to global impacts ranges between 6.8 and 8.0% (95% CI). For all other footprints, the spread between the 2.5th and 97.5th percentiles is less than 1 percentage point.

### Industry footprints

**Fig. 5:**
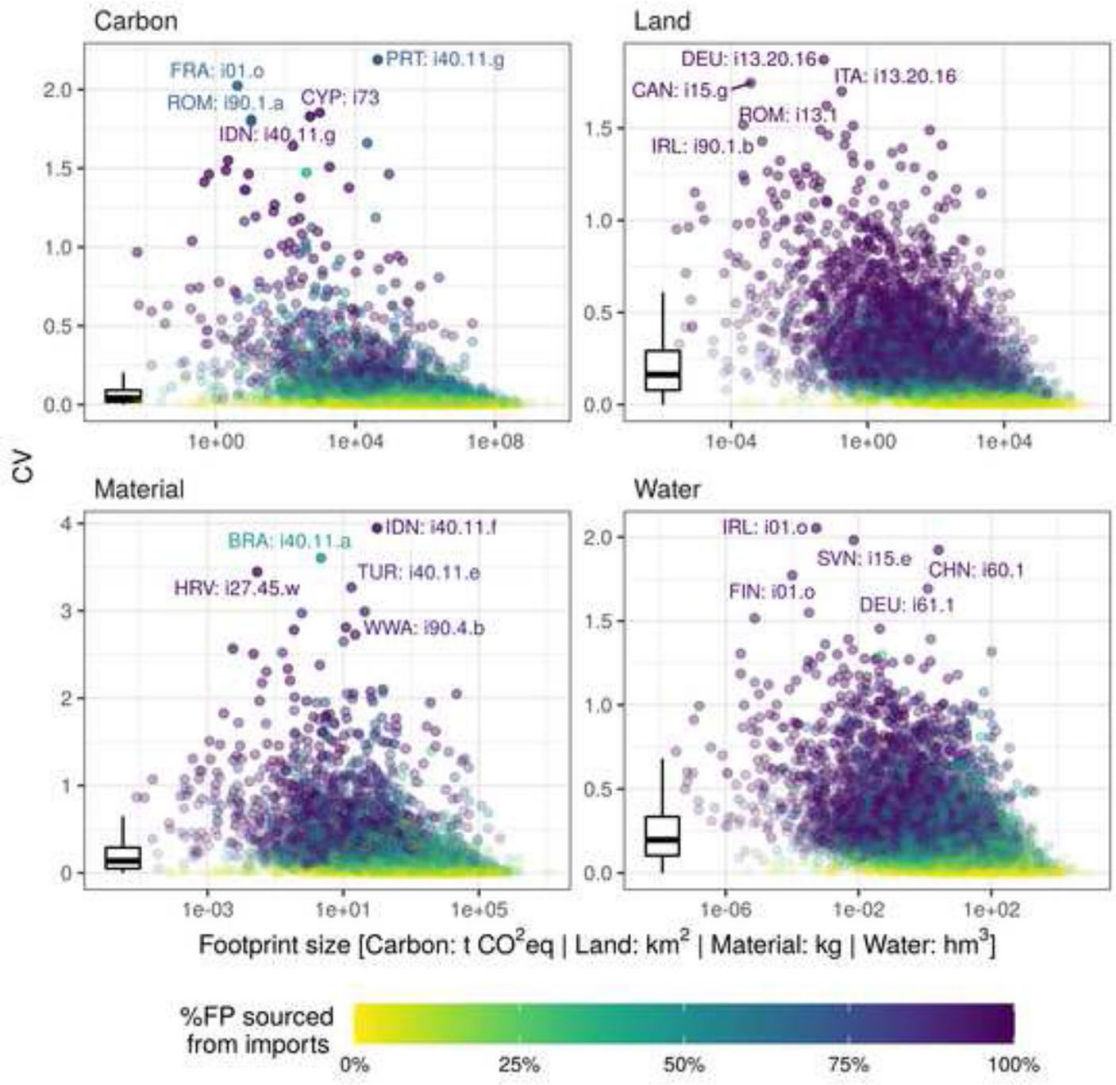
The relative variability (CV) of the industry footprints compared to their absolute size assuming proportional import shares. The 10 industries with the smallest non-zero footprint and all industries with a zero footprint are not shown. Boxplots show the distributions of the CVs across all industries with a non-zero footprint to facilitate comparison between the different types of footprints

For the industry footprints (Fig. 5) we see a similar pattern as for the national footprints. Considering *relative* variability (CV) the carbon footprints are less variable on average than land, material and water footprints (boxplots). Industries with a higher CV also have a higher import share. However, a high import share does not necessarily go hand in hand with a higher CV (compare in Additional file 2: Figure S3). With maximum CVs between 1.87 (land footprint) and 3.94 (material footprint) the variability at the level of industry sectors is significantly higher than at the regional/national level.

Industries of interest, i.e. with a relatively high CV and a relevant footprint size are—in the case of the carbon footprint—the ‘Electricity by biomass and waste’ (i40.11.g) sectors in Portugal (PRT) and India (IDN) with CVs of 2.19 and 1.66 respectively. In the case of land footprints, the sector ‘Other non-ferrous metal ores and concentrates’ (i13.20.16) in Germany (DEU, CV of 1.87) and Italy (ITA, CV of 1.70) stand out. Industries of interest in their material footprint are the Indian ‘Electricity by petroleum and other oil derivatives’ sector (IDN: i40.11.f) with a CV of 3.94, the Brazilian ‘Electricity by coal’ sector (BRA: i40.11.a) with a CV of 3.60 and the Turkish ‘Electricity by wind’ sector (TUR: i40.11.e) with a CV of 3.27. In the case of water footprints the sectors with a prominent role are the ‘Wool, silk-worm cocoons’ sectors (i01.o) in Ireland (IRL) and—less pronounced—Finland (FIN) with CVs of 2.05 and 1.77, respectively, the Chinese ‘Sale, maintenance, repair of motor vehicles, motor vehicles parts, motorcycles, motor cycles parts and accessories’ sector (CHN: i50.a) with a CV of 1.92, the Slovenian ‘Processing vegetable oils and fats’ sector (SVN: i15.e) with a CV of 1.98, and the German ‘Sea and coastal water transport’ sector (DEU: i61.1) with a CV of 1.69.

**Fig. 6:**
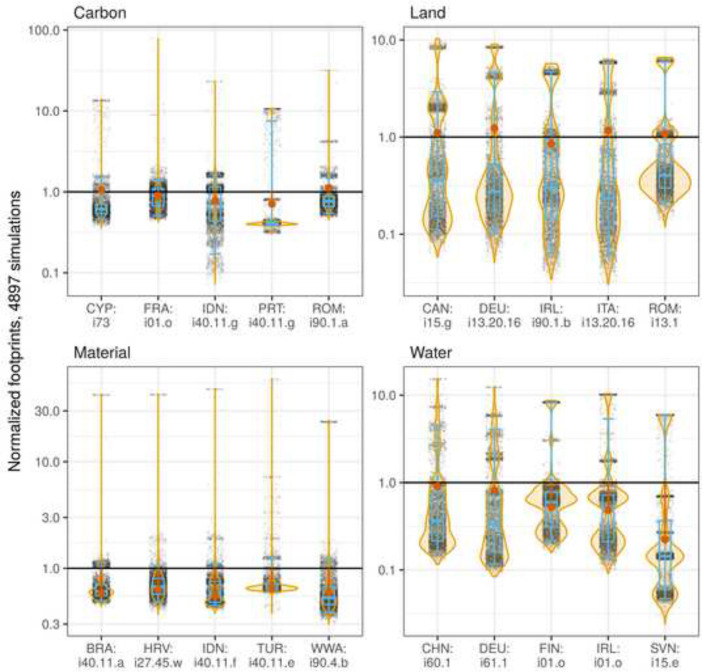
The sample distributions of the industry footprints exemplary for selected industries. The footprints where normalised by dividing each sample by the mean of all 4897 samples. The violin plots show the probability density and the range of all samples. The boxplots show the IQR (boxes), the sample’s median (vertical line) and the range from the 2.5th to the 97.5th percentile (whiskers). The red points show the footprints size assuming proportional import shares, also normalised by the mean of all samples

When taking a closer look at the distributions of the 4897 simulated industry footprints for aforementioned selected industries (Fig. 6), we see that most distributions are multi-modal. Additionally, most distributions have a positive skew. The most extreme upward deviation can be seen for the Portuguese ‘Electricity by biomass and waste’ (PRT: i40.11.g) sector’s carbon footprint with a 97.5th percentile of 1000%. Since the footprint calculated with the default version of EXIOBASE is even a bit below the sample mean, this finding implies that this sector’s carbon footprint can be more than 10 times higher than the current EXIOBASE version’s result. The largest downward deviation can be seen for the water footprint of the Italian ‘Mining of other non-ferrous metal ores and concentrates’ sector with a 2.5th percentile of 5%, implying a possible overestimation of this footprint by a factor of 20.

**Fig. 7:**
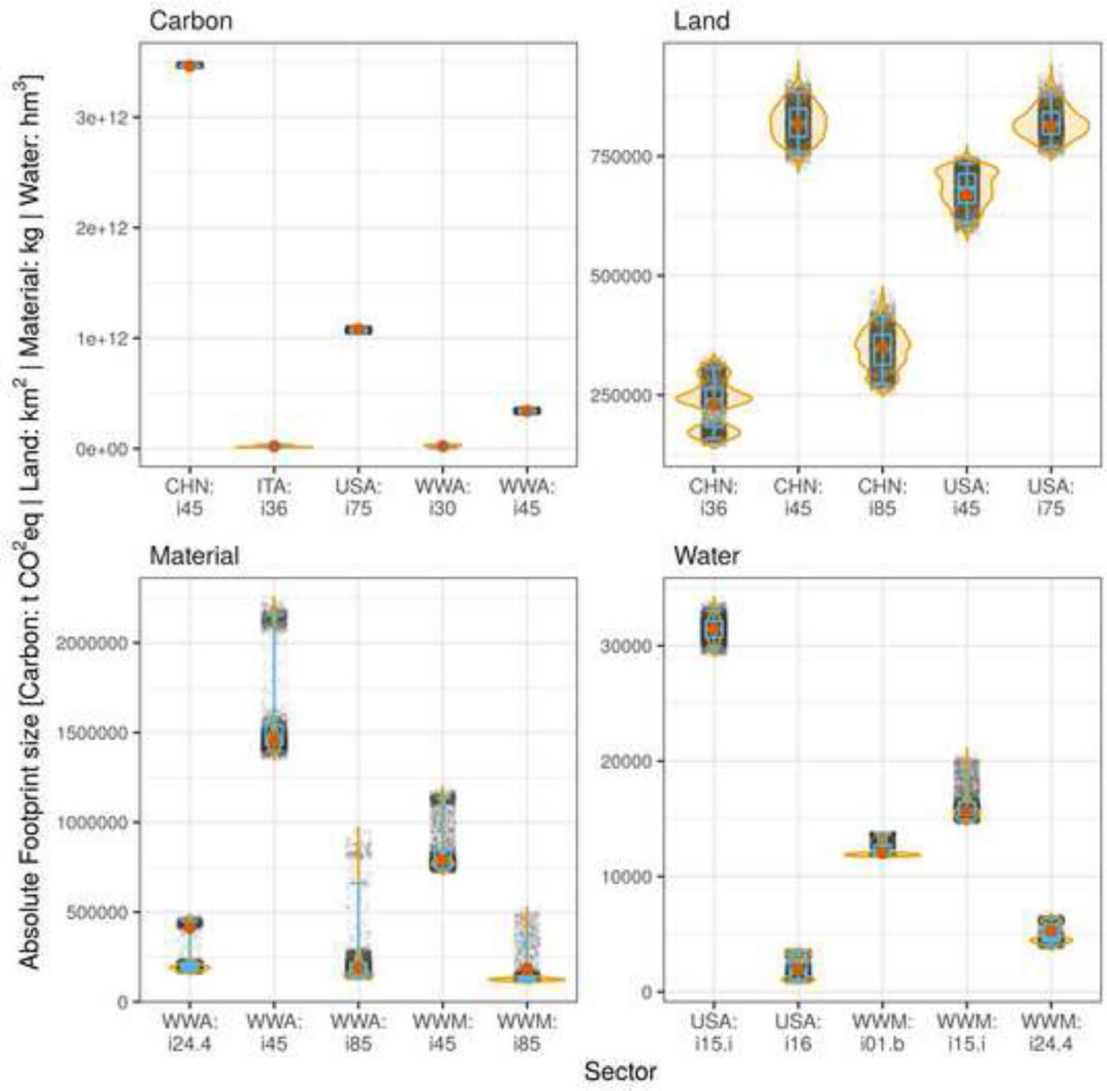
The sample distributions of the top five industries with the highest SD for each footprint category. The industries are ordered by their SD from left (high) to right (low). The red points show the footprints size assuming proportional import share

In contrast, other industry sectors stand out when looking at *absolute* variability (SD). Figure 7 shows the distributions of the top five industries with the highest SD for each footprint category. Again, industries are ordered by their SD from left (high) to right (low). The sector standing out the most is the construction sector (i45) with a high SD for WWA (carbon and material), China (carbon and land), USA (land) and RoW Middle East (WWM, material). The US ‘Public administration and defence; compulsory social security’ (USA: i76) stands out in terms of its land and carbon footprint. A high SD can also be seen for the ‘Health and social work’ sector (i85) in WWM and WWA (both material), and China (land). The ‘Manufacture of furniture; manufacturing n.e.c.’ sectors (i36) in Italy (carbon) and China (land) stand out, as does the ‘Processing of Food products nec’ sectors (i15.i) in WWM and the USA (both water). The widest spreads in absolute terms between the 2.5th and 97.5th percentiles we find are 51 Mt $\text {CO}_2\text{eq}$ emissions (USA: i75), 792 t of material extraction (WWA: i45), 160,000 $\text {km}^2$ of land use (CHN: i85), and 5000 $\text {hm}^3$ of water consumption (WWM: i15i).

## Discussion

Most published MRIO-based footprints come with no uncertainty by default. Given the high potential uncertainty due to several assumptions made in the process of compiling MRIO tables (Lenzen et al. 2010) this is problematic if these results are used for, or influence, decision making, as the robustness of the decision in relation to the footprint information used cannot be assessed due to the lacking uncertainty of the latter (Tukker et al. 2020). This piece quantifies the effect of one assumption underlying all global MRIOs—the proportionality assumption—on the four major environmental footprints of the different EXIOBASE regions and industries.

At the country level, the relative variability is in general below a coefficient of variation (CV) of 4%, except for the material, water and land footprints of highly trade-dependent and globally very small (Luxembourg, Malta, Latvia, Lithuania) and small economies (Belgium, Netherlands), and the RoW-region ‘Asia and Pacific’. More profound problems, however, are to be expected in the interpretation of the geographical origin of the national footprints (such as e.g. Schmidt et al. 2019; Tukker et al. 2014). Focusing on the footprints of regions with a globally considerable impact we find that the share of a region’s footprint in global impacts is relatively robust to a relaxation of the proportionality assumption. Only for the material footprint of the RoW-region ‘Asia and Pacific’ the spread between the 2.5th and 97.5th percentiles of the region’s share in global impacts is larger than 1 percentage point.

At the industry/product level we find the relative variability to be substantially higher as compared to national footprints. The footprints of 25% of the industry sectors covered by EXIOBASE that have a non-zero footprint^2^ show a CV above 10% (carbon footprint), above 30% (land and material footprint), and above 34% (water footprint), respectively. Some industry footprints, show a possible relative variability of 1000% or more indicating that assuming proportional import shares might lead to over- or underestimation of these footprints by a factor of 10 or more. In terms of absolute variability we find industry footprints with a spread between the 2.5th and 97.5th percentile of up to 51 Mt $\text {CO}_2\text{eq}$ emissions, 792 t of material extraction, 160,000 $\text {km}^2$ of land use, and 5000 $\text {hm}^3$ of water consumption. Our findings thus confirm that MRIO-based footprints at the industry/product level need to be treated with great care (Lenzen et al. 2010).

The greater relative variability at the industry level compared to the national level can be explained by the fact that national footprints are the sum of a multitude of industry footprints. Hence, at the more aggregated level of nations variability at the industry level will partly cancel out each other. Moreover, as elaborated in the introduction, for the industry footprints the proportionality assumption might already be problematic when only one condition is met (a large variation in the impact intensities between the industries of the exporting countries), while in the case of national footprints a second condition has to be satisfied (a large variation in the proportions of the importing industries that is consumed domestically).

The sample distributions of most industry footprints and some national footprints (Taiwan’s land footprint, WWA’s material footprint) are multi-modal, i.e. they have two or more local maxima in their density functions. One explanation could be that the variability of these footprints depends on the allocation of imports of only one (or few) products. Changing the allocation of only this (these few) product import(s) leads to a large change in the footprint (local maxima) while the allocation of all other global import flows has only a minor influence (little dispersion around the local maxima). Further research could zoom into these footprints, e.g. via a structural path analysis/decomposition, to determine the origin of the uncertainty introduced by the proportionality assumption (Lenzen 2003).

In general, we find that country and industry footprints with a high import share show a higher variability (compare also in Additional file 2: Figures S2 and S3). This finding is in line with Jiang et al. 2020 who found a high correlation between the percentage of the Chinese material footprint sourced from imports and the error of the footprint introduced by the proportionality assumption (Jiang et al. 2020). This relationship also seems to—at least partly—explain the overall lower variability of the carbon footprints (with relatively low import shares), compared to material, land, and water footprints (with import shares up to 100%). The finding that a high import share does not automatically lead to a high variability in the footprints suggests that a high import share is a necessary but not sufficient condition for a high uncertainty in national/industry footprints with regard to the proportionality assumption.

As elaborated in the introduction, from a theoretical perspective, MRIO results are only sensitive to changes in the allocation of an imported commodity if the impact intensities for the production of this commodity differ between exporting regions. We compared this between-region variability of the impacts intensities across all industry sectors between the four footprint categories to see if it also might explain parts of the gap in variability between carbon footprints and the remainder footprint categories. However, as it can be seen in Additional file 2: Figure S4, we find no significant signs of a lower between-region variability for carbon multipliers than for material, land and water multipliers.

With the algorithm we apply in this study we provide an upper boundary estimate of the uncertainty that might arise from the proportionality assumption in MRIO analysis. By only considering ‘extreme’ versions of how to allocate imports to target sectors, the sample distributions of the footprints (and all numbers derived from them) cannot be seen as the ‘true’ uncertainty distributions under the assumption that each possible allocation is evenly likely. However, we consider our study as a relevant contribution to the quantification of the maximum possible uncertainty that may arise from relaxing the proportionality assumption in MRIO.

One possible reason why the actual maximum uncertainty could be even *larger* than stated in this study, however, is that the uncertainty estimate is based on only 4897 iterations. Theoretically, with our allocation algorithm there exist almost infinite possibilities to construct ‘new’ $\varvec{Z}$ and $\varvec{Y}$ matrices. The computational limit of 4897 iterations is almost entirely owed to the calculation of the Leontief inverse which is—given the size of EXIOBASE—computational expensive even when solved as a system of linear equations (see also Çetinay et al. 2020).

In our analysis we use the industry-by-industry version of EXIOBASE. As mentioned in the methodology section, from a theoretical point of view it would be more conclusive to do the randomisation directly within the MRSUT framework, convert them to product-by-product MRIO tables and then calculate national and product footprints. However, this approach would require larger computing resources than we had available to obtain robust results. Moreover, we see no reason to believe that MRSUTs or product-by-product MRIOs will in general react differently to a relaxation of the proportionality assumption than the industry-by-industry version we used. Although it would certainly have a noteworthy impact on the footprints of some (few) industries/products.

In a next step, the question whether the inclusion of trade data from the BEC substantially decreases the uncertainty of the footprints could be answered quantitatively. Given the patchy and aggregate nature of the BEC data, however, compilers of highly disaggregated MRIOs such as EXIOBASE would still have to rely on strong assumptions. Another interesting research question would be the effect of the sectoral/regional resolution of a MRIO on the uncertainty introduced by the proportionality assumption. This could either be approached by conducting a similar analysis for other MRIOs having different sectoral/regional resolutions or using the same database but aggregating sectors/regions step-wise.

To contextualise our results we can compare the footprint variability we found to the variability found in other studies. Most studies to date dealing with the uncertainty in MRIO-based footprint analysis compared the results across databases (Rodrigues et al. 2018; Owen et al. 2014; Wieland et al. 2018; Wood et al. 2019; Moran and Wood 2014; Steen-Olsen et al. 2016). An exception is the work of Lenzen et al. (2010) that uses (inferred) standard deviations of the raw data to analyse how these uncertainties propagate to UK’s carbon footprint estimate. Almost all studies examine carbon footprints, only Giljum et al. (2019) focuses on material footprints, while for MRIO-based land and water footprint we could not find any study on uncertainty with a scope comparable to our work which would make it possible to compare our results with.

The most up-to-date study of the uncertainty of MRIO-based carbon footprints we are aware of is from Rodrigues et al. (2018). The authors analysed the uncertainty of national and product carbon footprints using the variability in the footprints between five different global product-by-product MRIO tables. At the level of national footprints they found CVs of 6% (USA), 9% (China) up to 16% for the Netherlands, and at the level of product footprints CVs between 10 and 213%. It should be noted, however, that they used product-by-product tables with a much lower sectoral resolution only distinguishing 17 product sectors compared to the 163 industry sectors we use in our analysis. A similar analysis with regard to material footprints was conducted by Giljum et al. (2019). They reported the variability of country material footprints as % difference in footprints between three different MRIO databases. To allow comparison to our results, we took their raw results and calculated the CV of selected country footprints. Taiwan, Slovakia and the Netherlands showed the highest variability in their material footprints with CVs of 40%, 37% and 25%, respectively. At the lower end range the US and the German material footprints with CVs of 6% and 2%, respectively.

When comparing these numbers with our results, we find that for national carbon footprints the variability caused by the proportionality assumption is only 1 to 4% of the inter-database variability found by Rodrigues et al. (2018). Although the maximum variability of industry footprints we found (219%) is in the same range compared to Rodrigues et al. (2018), due to the much higher sectoral resolution of our data, we can assume that when aggregating our results to an equivalent industry resolution the variability will be considerably reduced. Similarly, for national material footprints the variability caused by the proportionality assumption is only 0.2 to 2% of the inter-database variability found by Giljum et al. (2019). Hence, we conclude that the question of how the imported goods are distributed among the target sectors only leads to a low variability of the environmental footprints as compared to those assumptions made in the process of compiling environmentally-extended MRIOs that differ between individual databases, such as the source data, the use of the territorial versus residential principle, the chosen balancing algorithm or the breakdown/allocation of extensions (see also Tukker et al. 2020).

## Conclusion

A sound knowledge of the uncertainties in MRIO modelling is the basic prerequisite for the acceptance of MRIO-based results among policy makers. So far, the scientific literature on the uncertainty of MRIO-based environmental footprints, almost exclusively compared the results across databases. With our study we are adding a new perspective to this strand of literature by dealing with a source of uncertainty that affects all global MRIOs and has thus been ignored in inter-database comparisons: the import proportionality assumption. We quantified the global sensitivity of MRIO-based environmental footprint results to a relaxation of the proportionality assumption. We found that for carbon and material country footprints the variability caused by relaxing the proportionality assumption is only at most 4% of the inter-database variability found in previous studies.

However, as the variability of some industry and a few country footprints is too large to be ignored, and to help researchers that use MRIO to study environmental footprints at the national or sectoral level, we provide our main results in Additional files 3 and 4 in the form of .xlsx tables containing measures of the variability (SD, CV, 2.5th and 97.5th percentiles) of the environmental footprints of all regions and industries covered by EXIOBASE. With the help of these tables researchers can check if an industry/region that is important in their study ranks high, so that either the database can be improved through adding more details on bilateral trade, or the uncertainty can be calculated and reported.

## Supplementary Information

**Additional file 1.** A pseudo-code version of our algorithm we applied in this study.

**Additional file 2.** Relaxing the import proportionality assumption in multi-regional input-output modeling.

**Additional file 3.** Results national level. The data behind Figs. 3A and 4. The data of all individual model runs (needed to produce Fig. 3B can be get upon request from the author.

**Additional file 4.** Results industry level. The data behind Figs. 5 and 7. The data of all individual model runs (needed to produce Fig. 6) can be get upon request from the author.

**Additional file 5.** Country codes according to ISO 3166-1 alpha-3 except RoW regions.

## Data Availability

EXIOBASE V3.4 is available at https://exiobase.eu/. The R-code needed to reproduce the results of this article is available in the github repository https://github.com/simschul/import_proportionality under the commit ‘Publication version’ (90e65647967af7c87b75703813a1ce0230279f12). The results data supporting the conclusions of this article are included in Additional files 3 and 4.

## Footnotes

1 The number results from the maximum run time of 40 h allowed by the BWunicluster which was used for the analysis.

2 Note that EXIOBASE has a harmonised sector resolution i.e. the economy of each region is represented with the same set of industry sectors such that sectors which do not exist in a region (e.g. there is no Austrian paddy rice production) show up with zero inputs and outputs and thus a zero footprint

## References

[CR1] Brizga J, Feng K, Hubacek K (2017). Household carbon footprints in the Baltic states: a global multi-regional input–output analysis from 1995 to 2011. Appl Energy.

[CR2] Çetinay H, Donati F, Heijungs R, Sprecher B (2020). Efficient computation of environmentally extended input–output scenario and circular economy modeling. J Ind Ecol.

[CR3] Dietzenbacher E, Los B, Stehrer R, Timmer M, Vries G.d (2013). The construction of world input–output tables in the WIOD project. Econ Syst Res.

[CR4] Eurostat (2008). Eurostat manual of supply, use and input–output tables.

[CR5] Feenstra RC, Jensen JB (2012). Evaluating estimates of materials offshoring from US manufacturing. Econ Lett.

[CR6] Giljum S, Wieland H, Lutter S, Eisenmenger N, Schandl H, Owen A (2019). The impacts of data deviations between MRIO models on material footprints: a comparison of EXIOBASE, Eora, and ICIO. J Ind Ecol.

[CR7] Hardadi G, Buchholz A, Pauliuk S (2020). Implications of the distribution of German household environmental footprints across income groups for integrating environmental and social policy design. J Ind Ecol.

[CR8] Huang YA, Lenzen M, Weber CL, Murray J, Matthews HS (2009). The role of input–output analysis for the screening of corporate carbon footprints. Econ Syst Res.

[CR9] Ivanova D, Stadler K, Steen-Olsen K, Wood R, Vita G, Tukker A, Hertwich EG (2016). Environmental impact assessment of household consumption. J Ind Ecol.

[CR10] Jiang M, Liu L, Behrens P, Wang T, Tang Z, Chen D, Yu Y, Ren Z, Zhu S, Tukker A, Zhu B (2020). Improving subnational input–output analyses using regional trade data: a case-study and comparison. Environ Sci Technol.

[CR11] Lenzen M (2003). Environmentally important paths, linkages and key sectors in the Australian economy. Struct Change Econ Dyn.

[CR12] Lenzen M, Wood R, Wiedmann T (2010). Uncertainty analysis for multi-region input–output models—a case study of the UK’s carbon footprint. Econ Syst Res.

[CR13] Lenzen M, Moran D, Kanemoto K, Geschke A (2013). Building Eora: a global multi-region input–output database at high country and sector resolution. Econ Syst Res.

[CR14] Li JS, Chen B, Chen GQ, Wei WD, Wang XB, Ge JP, Dong KQ, Xia HH, Xia XH (2017). Tracking mercury emission flows in the global supply chains: a multi-regional input–output analysis. J Clean Prod.

[CR15] Majeau-Bettez G, Pauliuk S, Wood R, Bouman EA, Strømman AH (2016). Balance issues in input–output analysis: a comment on physical inhomogeneity, aggregation bias, and coproduction. Ecol Econ.

[CR16] Milberg W, Winkler DE (July 2010) Errors from the “proportionality assumption” in the measurement of offshoring: application to German labor demand. SSRN Scholarly Paper ID 1635800, Social Science Research Network, Rochester. 10.2139/ssrn.1635800

[CR17] Miller RE, Blair PD (2009). Input–output analysis: foundations and extensions.

[CR18] Moran D, Wood R (2014). Convergence between the Eora, WIOD, EXIOBASE, and OpenEU’S consumption-based carbon accounts. Econ Syst Res.

[CR19] Myhre G, Shindell D, Pongratz J (2014). Anthropogenic and natural radiative forcing.

[CR20] OECD (2020) BTDIxE bilateral trade in goods by industry and end-use. ISIC Rev. 4. https://stats.oecd.org/Index.aspx?DataSetCode=BTDIXE_I4. Accessed 21 July 2020

[CR21] Owen A, Steen-Olsen K, Barrett J, Wiedmann T, Lenzen M (2014). A structural decomposition approach to comparing MRIO databases. Econ Syst Res.

[CR22] Peters GP, Andrew R, Lennox J (2011). Constructing an environmentally-extended multi-regional input–output table using the GTAP database. Econ Syst Res.

[CR23] Puzzello L (2012). A proportionality assumption and measurement biases in the factor content of trade. J Int Econ.

[CR24] Rodrigues J, Marques A, Wood R, Tukker A (2016). A network approach for assembling and linking input–output models. Econ Syst Res.

[CR25] Rodrigues JFD, Moran D, Wood R, Behrens P (2018). Uncertainty of consumption-based carbon accounts. Environ Sci Technol.

[CR26] Schmidt S, Södersten C-J, Wiebe K, Simas M, Palm V, Wood R (2019). Understanding GHG emissions from Swedish consumption—current challenges in reaching the generational goal. J Clean Prod.

[CR27] Stadler K, Wood R, Bulavskaya T, Södersten C-J, Simas M, Schmidt S, Usubiaga A, Acosta-Fernández J, Kuenen J, Bruckner M, Giljum S, Lutter S, Merciai S, Schmidt JH, Theurl MC, Plutzar C, Kastner T, Eisenmenger N, Erb K-H, Koning Ad, Tukker A (2018). EXIOBASE 3: developing a time series of detailed environmentally extended multi-regional input–output tables. J Ind Ecol.

[CR28] Steen-Olsen K, Owen A, Barrett J, Guan D, Hertwich EG, Lenzen M, Wiedmann T (2016). Accounting for value added embodied in trade and consumption: an intercomparison of global multiregional input–output databases. Econ Syst Res.

[CR29] Steinmann ZJN, Schipper AM, Stadler K, Wood R, Koning Ad, Tukker A, Huijbregts MAJ (2018). Headline environmental indicators revisited with the global multi-regional input–output database EXIOBASE. J Ind Ecol.

[CR32] Tukker A, Bulavskaya T, Giljum S, De Koning A, Lutter S, Simas M, Stadler K, Wood R (2014) The global resource footprint of Nations—carbon, water, land and materials embodied in trade and final consumption calculated with exiobase 2.1, 2014. The Netherlands Organisation for Applied Scientific Research

[CR30] Tukker A, Koning Ad, Owen A, Lutter S, Bruckner M, Giljum S, Stadler K, Wood R, Hoekstra R (2018). Towards robust, authoritative assessments of environmental impacts embodied in trade: current state and recommendations. J Ind Ecol.

[CR31] Tukker A, Wood R, Schmidt S (2020). Towards accepted procedures for calculating international consumption-based carbon accounts. Clim Policy.

[CR33] Wiedmann T (2009). A review of recent multi-region input–output models used for consumption-based emission and resource accounting. Ecol Econ.

[CR34] Wiedmann TO, Lenzen M, Barrett JR (2009). Companies on the scale. J Ind Ecol.

[CR35] Wiedmann TO, Schandl H, Lenzen M, Moran D, Suh S, West J, Kanemoto K (2015). The material footprint of nations. Proc Natl Acad Sci.

[CR36] Wieland H, Giljum S, Bruckner M, Owen A, Wood R (2018). Structural production layer decomposition: a new method to measure differences between MRIO databases for footprint assessments. Econ Syst Res.

[CR37] Wood R, Moran DD, Rodrigues JFD, Stadler K (2019). Variation in trends of consumption based carbon accounts. Sci Data.

