# Supplementary material for: Relaxing the import proportionality assumption in multi-regional input–output modelling
Source: J Econ Struct. 2021 Oct 9;10(1):20. doi: 10.1186/s40008-021-00250-8 (PMC8549924; doi:10.1186/s40008-021-00250-8)
Supplement: Supplementary file 1 — Additional file 1. A pseudo-code version of our algorithm we applied in this study. [file 40008_2021_250_MOESM1_ESM.pdf]

Algorithm 1 Randomize matrix with given column-sums u and row-sums s

Require: s is vector
Require: u is vector

declare empty matrix to store the results: $\boldsymbol{T'} \leftarrow \underbrace{\left.\begin{array}{|c|c|c|}\hline 0 & \dots & 0 \\ \hline \vdots & \ddots & \vdots \\ \hline 0 & \dots & 0 \\ \hline \end{array}\right\}}_{length(u)} length(s)$

```
s' ← s
begin with country j ← 1
for i = industries in random order do
  u' ← u[i]
  while industry i still requires supply do
    if supply of country j ≤ use of industry i then
      entire supply of j is allocated to i: T'[j,i] ← s'[j]
      remaining need of i is updated: u' ← u' − s'[j]
      go to next country: j ← j + 1
    else
      industry i gets entire imported input from country j: T'[j,i] ← u'
      remaining supply of country j is updated: s'[j] ← s'[j] − u'
      industry i got its entire imported inputs: u' ← 0
    end if
  end while
end for
return T'
```
