# Supplementary material for: Relaxing the import proportionality assumption in multi-regional input–output modelling
Source: J Econ Struct. 2021 Oct 9;10(1):20. doi: 10.1186/s40008-021-00250-8 (PMC8549924; doi:10.1186/s40008-021-00250-8)
Supplement: Supplementary file 2 — Additional file 2. Relaxing the import proportionality assumption in multi-regional input-output modeling. [file 40008_2021_250_MOESM2_ESM.pdf]

Additional file 2 to: Relaxing the import proportionality assumption in multi-regional input-output modeling

Simon Schulte, Arthur Jakobs, Stefan Pauliuk

CONTENTS

1 Additional Results 2

2 Additional Analysis 3

1. ADDITIONAL RESULTS

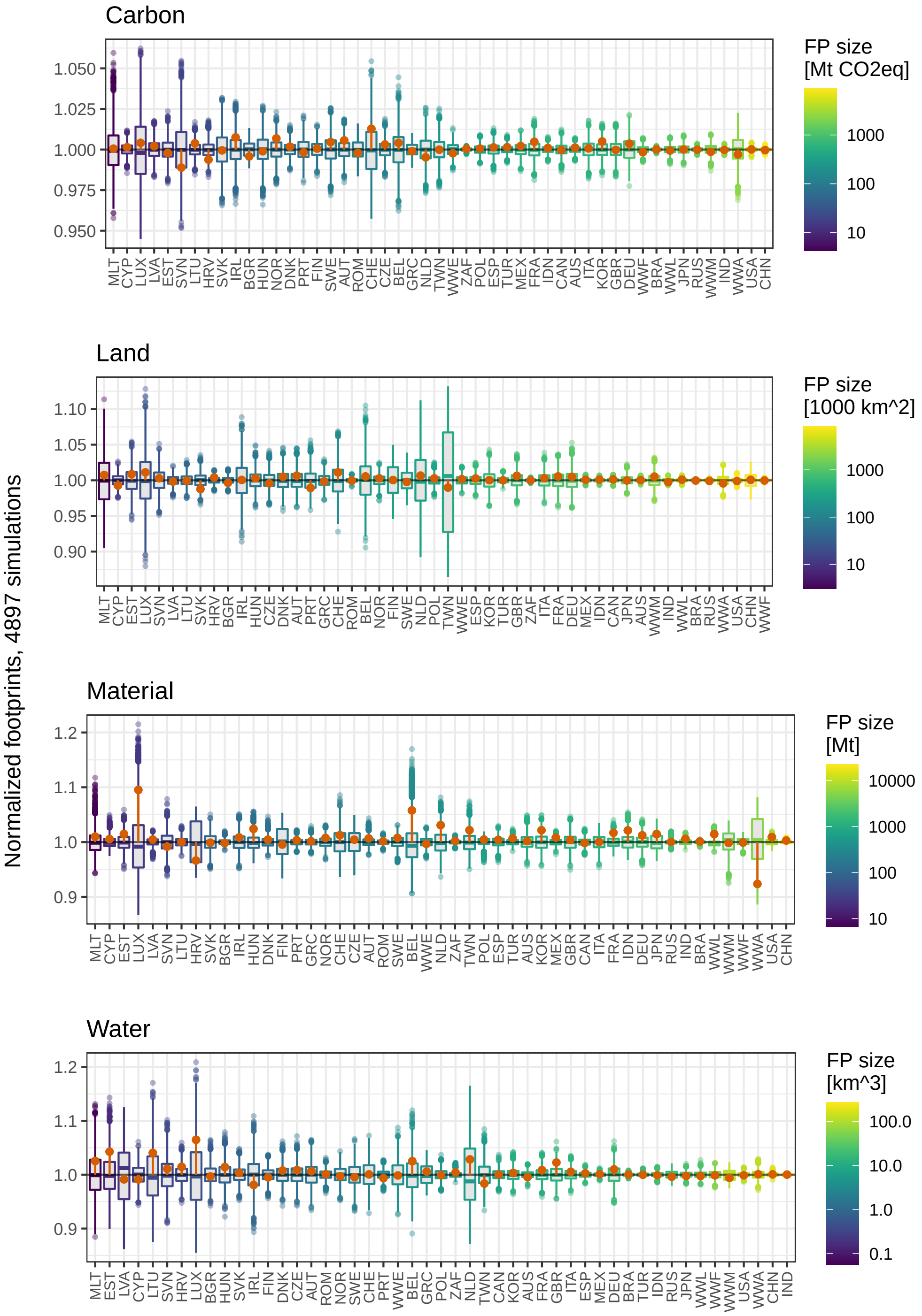

Fig. S1. Boxplots of the sample distributions of all national footprints ordered by their absolute size assuming proportional import shares. The footprints where normalised by dividing each sample by the mean of all 4897 samples. The boxplots show the inter-quartile range (IQR) where 50% of all samples are situated (boxes), the sample's median (vertical line) and the range covering 1.5 times the IQR (whiskers). The red points show the footprints size assuming proportional import shares, also normalised by the mean of all samples.

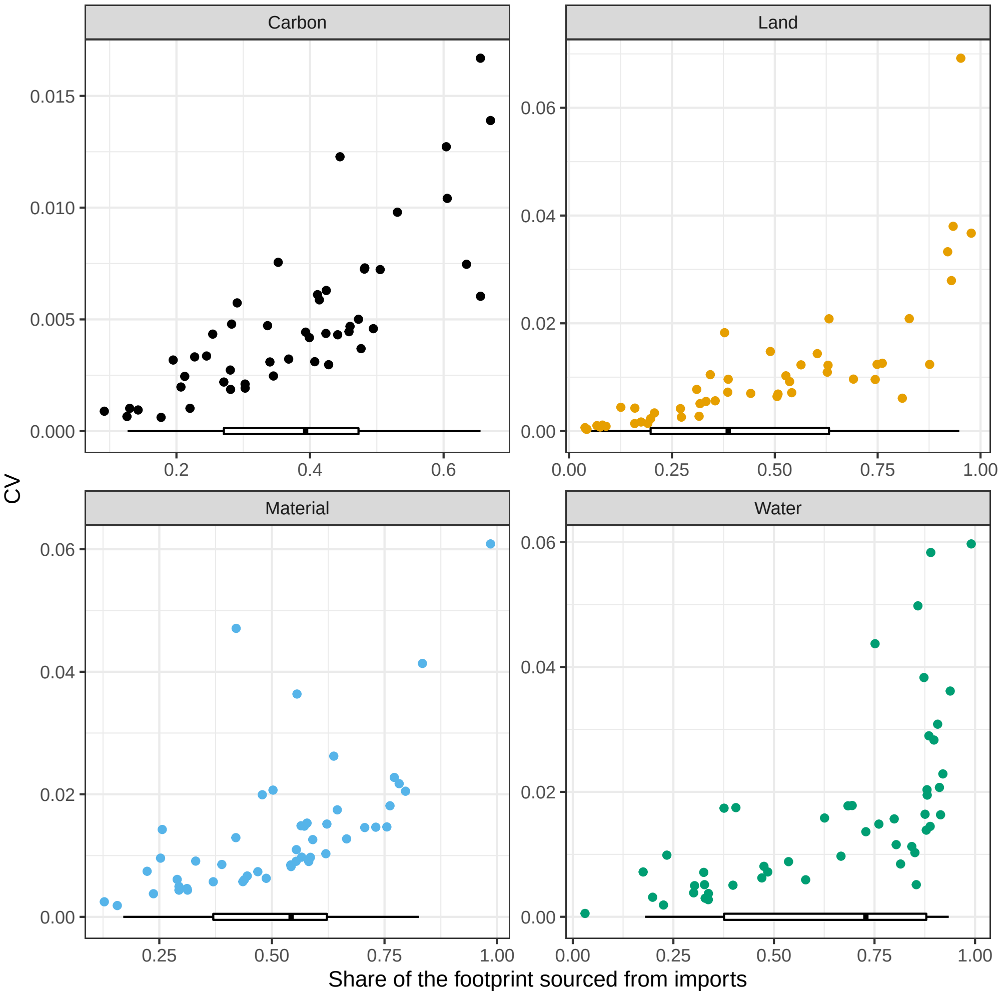

Fig. S2. The relationship between a national footprints' coefficient of variation (CV) and its share that is sourced from imports. The boxplots summarise the distributions of the CVs of the regions in the form of the IQR (boxes), the median (horizontal line) and the range from the 2.5th to the 97.5th percentile (whiskers).

2. ADDITIONAL ANALYSIS

As argued in the main article two factors could play a role in explaining the lower variability of carbon footprints compared with material, water and land footprints: (1) the lower import share of the former, and (2) the lower between-region variability of the carbon multiplier. For (1) we could find evidence that the share of industry/national carbon footprints that is sourced from imports is in general lower as compared to land/material/water footprints (see Figures S2 and S3). Argument (2), however, cannot be supported from a visual analysis as we show in Figure S4. For both measures of relative variability, CV and rMAD, we see at least no significant lower between-regions variability for carbon multiplier than for land/material/water multipliers. In contrast, the material multipliers show the lowest mean and median CV (both weighted by the industry sectors' shares of global impacts), and the water multipliers show the lowest mean and median rMAD (also weighted). Carbon multipliers rank in the middle for both variability measures and both location parameters.

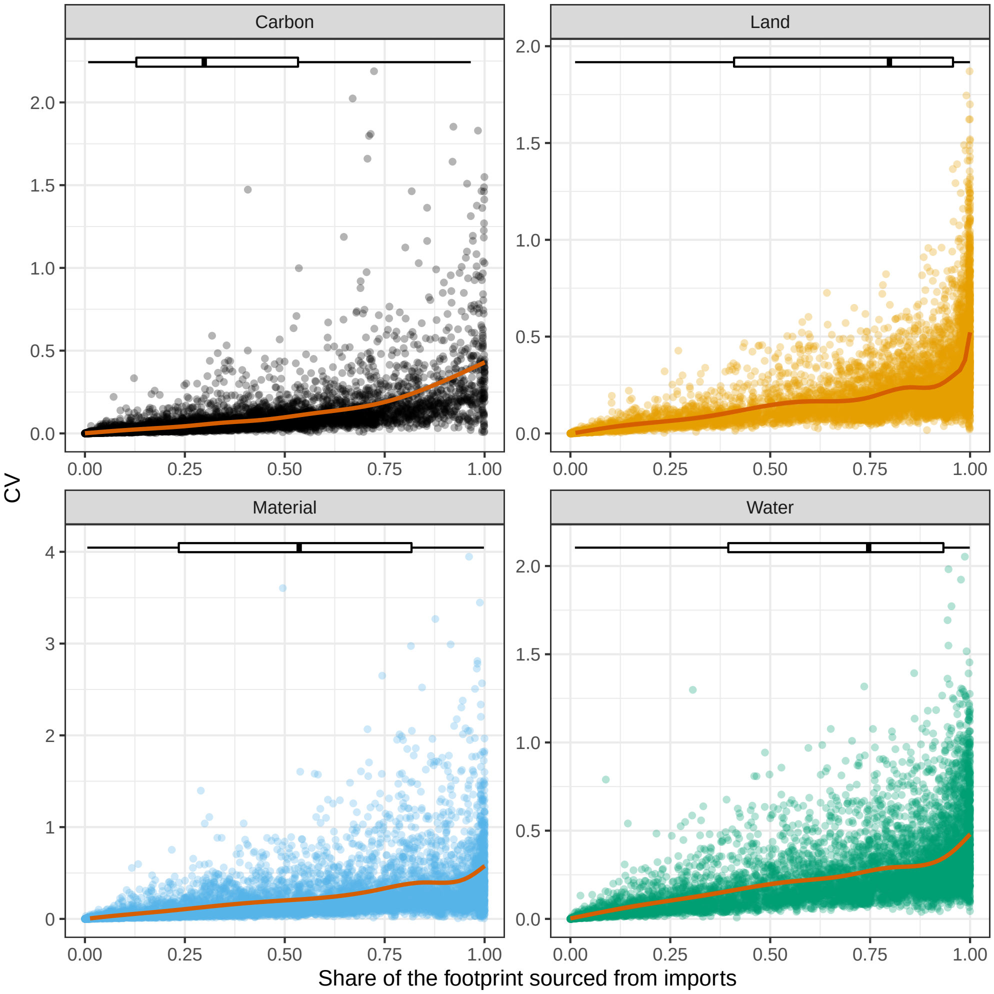

Fig. S3. The relationship between a industry footprints' coefficient of variation (CV) and its share that is sourced from imports. The red line shows a loess regression curve to reveal patterns in the presence of overplotting. The boxplots summarise the distribution of the CVs of the sectors in the form of the IQR (boxes), the median (horizontal line) and the range from the 2.5th to the 97.5th percentile (whiskers).

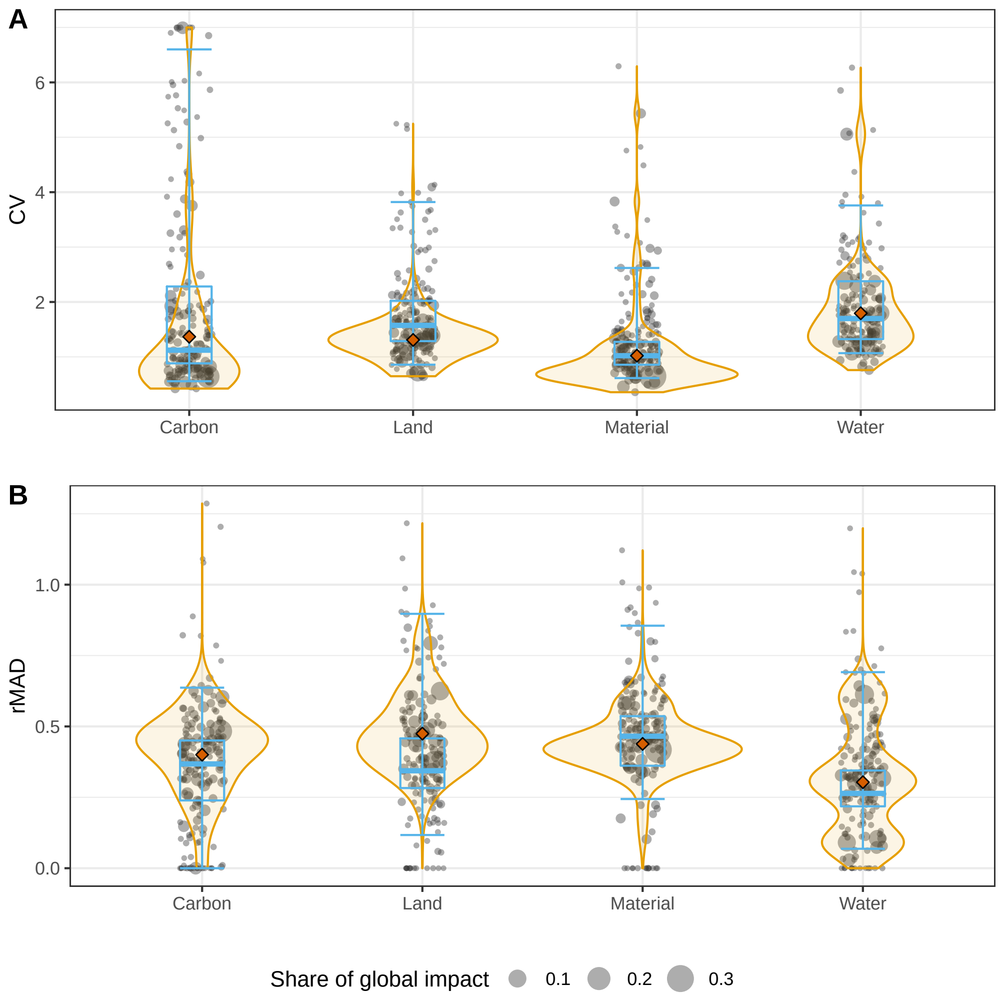

Fig. S4. The relative variabilities of the impact multipliers of the 163 industry sectors between regions measured with two different metrics: **A**: coefficient of variation (CV) and **B**: the relative mean absolute deviation (rMAD). The CV of the individual industry sectors are shown as grey data points, which vary in size depending on their share of the global impact. The violin plots, the boxplots, and the mean (red point) are based on weighted statistics with the share of global impacts as weighting parameter. The violin plots show the weighted probability density and the range of all samples. The boxplots show the weighted inter-quartile range (IQR) where 50% of all samples are situated (boxes), the sample's weighted median (horizontal line) and the range from the weighted 2.5th to the weighted 97.5th percentile (whiskers).
